# The ARIC predictive model reliably predicted risk of type II diabetes in Asian populations

**DOI:** 10.1186/1471-2288-12-48

**Published:** 2012-04-13

**Authors:** Calvin Woon-Loong Chin, Elian Hui San Chia, Stefan Ma, Derrick Heng, Maudrene Tan, Jeanette Lee, E Shyong Tai, Agus Salim

**Affiliations:** 1 Department of Cardiology, National Heart Centre, Singapore, Singapore; 2 Epidemiology and Disease Control Division, Ministry of Health, Singapore, Singapore; 3 Office of Clinical Research, SingHealth, Singapore, Singapore; 4 Department of Medicine, Yong Loo Lin School of Medicine, National University of Singapore, Singapore, Singapore; 5 Saw Swee Hock School of Public Health, National University of Singapore, MD3, 16 Medical Drive, Singapore 117597, Singapore

## Abstract

**Background:**

Identification of high-risk individuals is crucial for effective implementation of type 2 diabetes mellitus prevention programs. Several studies have shown that multivariable predictive functions perform as well as the 2-hour post-challenge glucose in identifying these high-risk individuals. The performance of these functions in Asian populations, where the rise in prevalence of type 2 diabetes mellitus is expected to be the greatest in the next several decades, is relatively unknown.

**Methods:**

Using data from three Asian populations in Singapore, we compared the performance of three multivariate predictive models in terms of their discriminatory power and calibration quality: the San Antonio Health Study model, Atherosclerosis Risk in Communities model and the Framingham model.

**Results:**

The San Antonio Health Study and Atherosclerosis Risk in Communities models had better discriminative powers than using only fasting plasma glucose or the 2-hour post-challenge glucose. However, the Framingham model did not perform significantly better than fasting glucose or the 2-hour post-challenge glucose. All *published* models suffered from poor calibration. After recalibration, the Atherosclerosis Risk in Communities model achieved good calibration, the San Antonio Health Study model showed a significant lack of fit in females and the Framingham model showed a significant lack of fit in both females and males.

**Conclusions:**

We conclude that adoption of the ARIC model for Asian populations is feasible and highly recommended when local prospective data is unavailable.

## Background

Type 2 diabetes mellitus (T2DM) and its associated complications have imposed a massive burden on public health care systems. Intensive lifestyle modification has been shown to effectively prevent or delay the development of T2DM [1-3]. Although effective, these interventional programs do incur some health care costs. In the Diabetes Prevention Program in the United States, the societal cost of lifestyle intervention was $3,540 more per individual than the placebo group over 3 years [4]. This cost would prevent one case of diabetes for every 6.9 persons treated with lifestyle modification programs over 3 years [1]. The number of persons that needs to be treated to prevent one case of T2DM--and thus the cost-effectiveness of the program--is highly dependent on the absolute risk of developing T2DM in the population undergoing the intervention. Thus, some form of risk assessment, with intervention targeted at those at highest risk, is critical to ensure the cost-effectiveness of such programs.

To date, randomized clinical trials have been relying on the diagnosis of impaired glucose tolerance (IGT) to selectively identify a population at high risk of T2DM. This diagnosis requires the administration of an oral glucose tolerance test (OGTT), which is inconvenient to both the clinician administering the test and the patient, leading to some reluctance in using it. Recognizing this reluctance, the American Diabetic Association (ADA), in 1997, encouraged the use of fasting plasma glucose (FPG) rather than the OGTT for the diagnosis of T2DM [5]. Levels of FPG between 'normal' and 'diabetic' were classified as impaired fasting glucose (IFG), a category analogous to IGT. However, IFG may not identify all individuals with the same degree of risk as those with IGT [6,7].

Several studies have shown that multivariable predictive functions using variables collected by medical history and fasting blood tests perform as well, if not better, than the 2-hour post-challenge glucose (2hPG) in identifying individuals at high or low risk of developing T2DM [8-10], thereby obviating the need for an OGTT. However, the broad utility of these functions is limited by the relative lack of studies that have assessed the ability of these functions to accurately predict the absolute risk of developing T2DM, which is important for the planning of interventional strategies for the prevention of T2DM. Ideally, a population-specific predictive function should be used for risk prediction [8,10,11]. However, suitable prospective data that would facilitate the development of these predictive functions is not always available, particularly in developing countries in the Asia-Pacific region, where the rise in the prevalence of T2DM is expected to be the greatest in the next several decades [12]. In this situation, applying predictive functions developed in an external population seems to be a reasonable alternative. However, differences in the average levels of risk factors and their effect sizes, length of follow-up and baseline incidence rates will likely complicate the evaluation. In particular, it is quite possible to underestimate or overestimate the actual risk due to these differences. Furthermore, there are several risk functions available [8,13,14] and it is not clear which risk function would perform best when applied to the Asian populations.

In order to determine the viability of adopting an externally-developed predictive function for T2DM to the Asian populations, we evaluated the performance of three multivariate predictive functions for T2DM risk: the San Antonio Health Study (SAHS) model [8], the Atherosclerosis Risk in Communities Study (ARIC) model [13] and the Framingham Offspring Study (FRAM) model [14]. These three models were chosen because a recent study had shown that they had excellent discriminative and calibration qualities when applied to multiethnic cohorts in the United States [15]. However, in order to use these functions in Asian populations, further evaluation of these functions is needed. The functions were evaluated using data from Singaporean Chinese, Malay and Asian Indians who were followed up as part of the Singapore Prospective Study Program (SP2) in the 1992 National Health Survey (NHS-92).

## Methods

### The 1992 Singapore National Health Survey (NHS-92) Cohort

**Figure 1:**
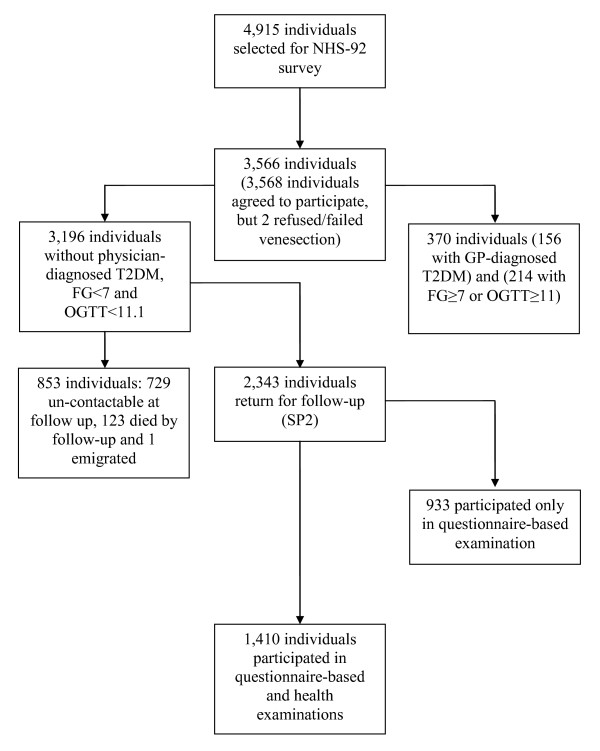
**Flowchart of NHS-92 subjects**.

The 1992 Singapore National Health Survey (NHS-92) was a cross sectional study carried out between September and November 1992 to determine the risk factors for major non-communicable diseases in Singapore. The results and methodology have been previously reported [16]. Systematic sampling from a national household database, followed by disproportionate stratified sampling by ethnic groups, was used to select the sample for the survey. The two minority ethnic groups, Malays and Asian Indians, were over-sampled to obtain an ethnic distribution of approximately 60% Chinese, 20% Malays and 20% Asian Indians to ensure sufficient numbers for statistical analysis. A total of 4,915 individuals were invited to join the study. Out of this, 3,568 individuals (response rate = 72.6%) agreed to enrol in the current study (Figure 1). Response rates were significantly different across ethnic groups, with the Chinese having a significantly higher response rate compared to other ethnic groups [16].

The individuals who agreed to participate in NHS-92 survey completed an interviewer-administered questionnaire and underwent a baseline physical examination, which included anthropometric measurements (including height, weight and waist circumference), blood pressure, and measurements of fasting plasma glucose and lipids. Height (to the nearest millimeter) was recorded in all subjects without shoes, and weight (in kilograms) was measured, with subjects in light clothing, using electronic weighing scales (SECA model 220). Waist (defined as the narrowest part of the body below the costal margin) and hip (defined as the widest part of the body below the waist) measurements were also taken; fasting blood samples including serum lipids (10 ml plain tubes) and glucose (2 ml fluoride oxalate tubes) were taken from 3,566 out of 3,568 subjects after an overnight fast of 10 hours (two subjects failed or refused venesection, and were thus excluded). OGTT was given to 3,435 subjects who were not on oral hypoglycemic medications or insulin (a completion rate of 96.3%). Plasma glucose and lipid measurements were performed on the same day as blood collection.

### Follow-up of NHS-92 participants

At baseline, there were 156 subjects with a previous diagnosis of T2DM. A further 214 of the NHS-92 subjects were diagnosed with diabetes mellitus at baseline, which was defined as FPG ≥ 7.0 mmol/L or 2hPG ≥ 11.1 mmol/L. These subjects were excluded, leaving 3,196 individuals in the NHS-92 cohort. Between 2004 and 2007, we conducted a study to follow up these individuals. The follow-up study consisted of an interviewer-administered questionnaire and a health screening, which included physical examination and blood tests. Ethics approval was obtained from the two Institutional Review Boards (National University of Singapore and Singapore General Hospital) prior to commencement of the study. Informed consent was obtained from the participants. The participants who were deceased at the time of follow-up (as shown by data-linkage with the Registry of Births and Deaths) were excluded from the study. The participants, whose contact details were provided by the Ministry of Home Affairs using their unique National Registration Identification Card number, were contacted first by mail, then by phone. Trained field interviewers conducted a home visit if there was no response or no available phone number. A minimum of three home visits at different times of the day (including a weekday and a weekend) were attempted before a participant was deemed unreachable.

Out of 3,196 individuals at baseline, 123 individuals had died, one emigrated and 729 individuals were not contactable at follow-up (Figure 1). The remaining 2,343 individuals were surveyed using interviewer-administered questionnaires to gather information on demographics and medical history during the home visits. Participants who returned were older, slightly more obese and less likely to be Chinese (Table 1). Those who did not return had lower baseline FPG and higher serum high density lipoprotein (HDL), and were less likely to have a family history of T2DM (Table 1). Subsequently, all the participants were invited to attend a health examination following a 10-hour overnight fast. A total of 1,410 individuals agreed to attend the health examination (Figure 1). Fasting blood specimens of these individuals were collected in fluoride oxalate tubes and sent in containers maintained at a temperature of 4°C for same-day analysis at the Referral Laboratory of the National University Hospital, Singapore, an American College of Pathologists-accredited laboratory. FPG was measured by enzymatic methods (ADVIA 2400, Bayer Diagnostics), with the blood collected in fluoride oxalate tubes. No OGTT was administered at follow-up. The outcome of interest was incident T2DM at follow-up, defined as having a FPG ≥ 7.0 mmol/L at follow-up or a physician diagnosis of T2DM.

**Table 1:** Baseline characteristics of participants without diabetes in the National Health Survey 1992

Baseline Characteristics	National Health Survey 1992 Total number of participants without diabetes at baseline, N = 3,196					
	
	**A**. Participants who did the survey and FPG during follow-up and have complete baseline information n = 1,401	**B**. Participants who did only the survey during follow-up n = 942	**C**. Participants who did not return for follow-up n = 729*	*P *value		
				
				A vs. B	A vs. C	(A+B) vs. C
**Age, years**	36.14 ± 10.84	36.59 ± 13.00	34.01 ± 12.32	0.36	< 0.001	< 0.001

**Males, n (%)**	669 (47.8)	450 (47.6)	333 (45.7)	0.93	0.36	0.34
**Females, n (%)**	732 (52.2)	496 (52.4)	396 (54.3)	0.93	0.36	0.34

**Chinese, n (%)**	924 (66.0)	592 (62.6)	517 (70.9)	0.09	0.02	0.002
**Malay, n (%)**	245 (17.5)	206 (21.8)	121 (16.6)	0.01	0.61	0.11
**Asian Indian, n (%)**	232 (16.5)	148 (15.6)	91 (12.5)	0.55	0.01	0.01

**BMI (kg/m2)**	22.80 ± 3.85	22.92 ± 4.11	22.41 ± 4.07	0.47	0.03	0.009

**FPG (mmol/L)**	5.35 ± 0.44	5.38 ± 0.49	5.27 ± 0.43	0.12	< 0.001	< 0.001

**HDL (mmol/L)**	1.26 ± 0.31	1.27 ± 0.31	1.31 ± 0.31	0.44	< 0.001	0.002

**SBP (mmHg)**	114.45 ± 14.58	117.15 ± 17.30	114.89 ± 16.13	< 0.001	0.52	0.34

**Positive family history of T2DM, n (%)**	497 (35.5)	237 (25.1)	175 (24.0)	< 0.001	< 0.001	< 0.001

* Excluded deceased individuals and individuals who moved out of the country

### Statistical approaches

#### Model development

We excluded 370 individuals who had been previously diagnosed with T2DM by a physician or were diagnosed with T2DM (FPG ≥ 7.0 mmol/L or 2hPG ≥ 11.1 mmol/L) at baseline. A further 853 individuals who had died, emigrated or were unreachable at follow-up were also excluded. This left us with 2,343 participants who returned for follow-up, out of which 1,410 individuals attended the health examination (Figure 1) and therefore had their FPG measured at follow-up. However, nine individuals with FPG measurement at follow-up did not have complete baseline information needed to build and evaluate the predictive functions. Hence, the final number of subjects included for statistical analysis was 1401.

For each *published* multivariate function, logistic regression was used to estimate the *local* version of the function. By *local* version of the function, we mean a model that used the same set of predictors as the corresponding *published* model, but whose coefficients were estimated using data from the NHS-92 cohort. In addition to the *local* version of the published multivariate models, two *local* models that used only FPG and 2hPG as predictors were estimated. In the SAHS and ARIC models, a dichotomous ethnicity variable was used as part of the predictive function. To enable application of these two models to the Singaporean population, individuals in the NHS-92 cohort were categorized into two categories, with Chinese ethnicity as the reference category, and non-Chinese ethnicity to include Malays and Asian Indians. The choice of Chinese as the reference category was motivated by the fact that prior research had found that the Chinese population in Singapore had lower incidence rates of T2DM than the Malays and Asian Indians. This choice is comparable to the choice in ARIC and SAHS models, where ethnic groups with lower incidence rates were chosen as the reference group for the studies. For the *local* version of the ARIC and SAHS models, we initially had two separate dummy variables for Malays and Asian Indians, but these two dummy variables were combined when the goodness of fit for model with one combined race variable is better than using two separate race variables.

For all multivariate models, between-population heterogeneity was investigated by examining the interaction terms between covariates in the model and ethnicity. Interaction terms found to be statistically significant using likelihood ratio test (*P *< 0.05) were subsequently added to the *local* models.

Since the *local* models were estimated using only 1,401 out of 2,343 subjects who returned for follow-up, we assessed the impact of excluding nine individuals with incomplete baseline information and 933 individuals with missing FPG measurements at follow-up. We did this by performing multiple imputation on the incomplete observations. For each of the 942 subjects with missing baseline information or FPG at follow-up, 20 imputed values are generated for the missing information using fully conditional specification (FCS) imputation [17,18]. The following baseline variables are used in the regression model for predicting FPG levels at follow-up (imputer model): age, gender, race, systolic blood pressure, body mass index (BMI), high density lipoprotein (HDL), low density lipoprotein (LDL), FPG at baseline, family history of T2DM, hip circumference, waist circumference, waist-hip ratio, creatinine levels, triglyceride and insulin resistance. To obtain the estimates under multiple imputation, logistic regression was fitted to each of the 20 imputed datasets separately. The multiple imputation estimates were obtained as the average estimates across the imputed datasets and the standard errors of the estimates were computed using Rubin's formula by taking into account both within and between imputation variance.

The probability that an individual developed T2DM during the follow-up period was estimated using the following formula: *p = 1/(1 + e^-y^)*, where *p *is probability of incident diabetes *y *is the logistic regression function.

### Model comparisons

#### Discrimination power

The predictive functions were compared in terms of the discriminative power and calibration quality. To assess the discrimination power of a predictive function, we used the area under the receiver operating characteristic curves (AUC). The AUC represents an estimate of the probability that a model assigns a higher risk to those who develop T2DM during the follow-up period than to those who do not. When comparing the AUC of two different models, we used the method by DeLong et al. [19], with *P *< 0.05 to indicate statistical significance.

We also used a more sensitive method called Net Reclassification Index (NRI) [20] for comparing the discrimination power of the *published* models. For NRI calculation, the predicted probabilities from each model were classified into 0-10%, 10.1-20%, 20.1-30%, 30.1-40%, 40.1-50% and > 50% risk categories. A better reclassification for a case takes place whenever a case is placed at a higher risk category, while for non-cases, the opposite is true.

As we were interested in the applicability of the *published* model where local prospective data was not available, we compared the performance of the predictive models in the following manner: for each multivariate model, the discriminative ability of the *published* model was compared to the *local* model. The objective here was to assess the 'gap' between the *published* model and the best model developed from local data, had the data been available. The *published* models were also compared to the *local* models that used only 2hPG or FPG as a predictor. The objective of this last comparison was to assess the potential for using externally-developed predictive model as an alternative to the 2hPG or FPG for identifying high-risk individuals. Finally, comparisons between the *published* models were made to examine which *published* model had the best performance and thus could potentially be recommended for use in Asian populations.

### Calibration quality

The Hosmer-Lemeshow goodness of fit test was used to assess the calibration quality of the various predictive models. For all models, the predicted and observed number of events in each quintile of estimated probability were calculated and the test statistic was compared to the χ^2 ^statistic with 3 degrees of freedom. Values of the test statistic exceeding 11.5 (*P *< 0.01) implied a significant lack of calibration [21]. For the *published* models, we also performed the recalibration step (see Appendix A) to assess the extent to which the lack of calibration was caused simply by differences in baseline incidence rates and average risk factors between the two populations. To perform the recalibration, we needed to estimate from the local population the average values for each risk factor and the probability that a subject with the average risk factors survived the study period without being diagnosed with T2DM. The Hosmer-Lemeshow statistics were computed both before and after recalibration to assess the effect of recalibration on the performance of the externally derived predictive function.

All statistical analyses were carried out using R 2.9.0 http://www.r-project.org and STATA version 10 (Stata Corporation, College Station, TX).

## Results

For all *local* predictive models, no interactions between ethnicity and other covariates were found to be significant, so no interaction term was added to any of the *local* predictive models. *local* ARIC and SAHS models with one combined race variable for Malays and Asian Indians had better model fit than their counterparts with two separate race variables. For *local* SAHS models, the Akaike Information Criterion (AIC) for models with one and two race variables were 588.6 and 590.6, respectively. For *local* ARIC models, the AIC were 587.9 and 589.8, respectively.

Table 2 shows comparisons of the coefficients of the three multivariate predictive functions that were estimated using the NHS-92 cohort with the *published* coefficients from the SAHS, ARIC and Framingham models.

**Table 2:** Comparison of *published* and locally-estimated multivariate predictive functions

	**SAHS Model**		**ARIC Model**		Framingham Model*	
	
**Risk factor at baseline**	**locally-estimated (95% CI)**	***Published***	**locally-estimated** **(95% CI)**	**Published**	**locally-estimated (95%CI)**	**Published**
	
Age (years)	-***0.004 (-0.026; 0.018)***	***0.028***	***-0.023 (-0.047; 0.000)***	***0.0173***	NA	NA
Gender	***0.098 (-0.391; 0.588)***	***0.661***	NA	NA	NA	NA
Ethnicity	0.646 (0.186; 1.107)	0.412	0.677 (0.214; 1.140)	0.4433	NA	NA
FPG (mg/dl)	***0.119 (0.090; 0.148)***	***0.079***	***0.120 (0.090; 0.149)***	***0.0880***	NA	NA
IFG	NA	NA	NA	NA	1.795 (1.259;2.331)	1.98
SBP (mmHg)	0.015 (0.000; 0.030)	0.018	0.015 (0.000; 0.029)	0.0111	0.843 (0.372;1.315)	0.50
Triglyceride(mg)	NA	NA	***0.000 (-0.001;0.001)***	***0.0027***	0.695 (0.232;1.158)	0.58
HDL (mg/dl)	-0.033 (-0.058;-0.008)	-0.039	-0.027 (-0.053;-0.001)	-0.0122	0.498 (0.032;0.965)	0.94
BMI (kg/m^2^)	***0.154 (0.097; 0.212)***	***0.070***	NA	NA	NA	NA
BMI (overweight)	NA	NA	NA	NA	***1.206 (0.728;1.683)***	***0.30***
BMI (obese)	NA	NA	NA	NA	1.574 (0.881;2.267)	0.92
Waist (cm)	NA	NA	***0.071 (0.046; 0.096)***	***0.0273***		NA
Height (cm)	NA	NA	-0.044 (-0.072;-0.016)	-0.0326		NA
Family history of T2DM	0.447 (-0.008;0.903)	0.481	0.473 (0.018; 0.928)	0.4981	0.548 (0.111;0.986)	0.57

Bold and italicized fonts: Estimates from local models whose 95% confidence intervals do not overlap with the published estimates, i.e. we assumed that the published estimates were the true values in the hypothesis testing

*Categorical variables are used for the Framingham model with the following rule: fasting glucose level: (1 = 100 - 125 mg/dL, 0 ≤ 100 mg/dL), overweight (1 = BMI ≥ 25 & BMI < 30, 0 = otherwise), obese (1 = BMI ≥ 30, 0 = otherwise), low high density lipoprotein (HDL) (1 = HDL < 40 mg/dL in men or < 50 mg/dL in women, 0 = otherwise), high triglyceride level (1 ≥ 150 mg/dL, 0 = otherwise), elevated blood pressure (systolic blood pressure ≥ 130 or diastolic blood pressure ≥ 85 mm Hg, 0 = otherwise)For the SAHS model, the 95% confidence intervals of the coefficients estimated from the NHS-92 cohort included the *published* SAHS coefficient for all but four risk factors: age, gender, FPG at baseline and BMI; the coefficients for age and gender were greater for the *published* SAHS model than the locally-estimated coefficients. In contrast, the locally estimated coefficients for FPG and BMI were larger than the *published* coefficients.

For the ARIC model, the 95% confidence intervals of the following risk factors did not include the *published* effect size from the original study: age, fasting FPG at baseline, waist circumference and triglyceride.

For the Framingham model, all locally-estimated effect size agreed well with the *published* ones, except for the effect of overweight, which was found to be significantly higher in our *local* model.

However, at this stage, we did not know to what extent the estimates from the *local* models had been influenced by the exclusion of 942 subjects without FPG measurement at follow-up or due to incomplete baseline information.

Table 3 shows the multiple imputation estimates for the three *local* models. Interestingly, after taking into account the dropout effects, the discrepancies between the *local* and *published* models were less startling. In particular, for the SAHS and ARIC models, the discrepancies in terms of FPG and measures of adiposity (BMI or waist circumference) were no longer statistically significant. There were, however, significantly smaller age and gender effect sizes when compared to the *published* models. With the Framingham model, we found a significantly larger effect of overweight, quite possibly because a Caucasian definition of overweight (BMI ≥ 25) had been used instead of the WHO recommendation for Singapore, which used a cut-off of BMI ≥ 23 to define overweight individuals [22].

**Table 3:** Comparisons of *published *and locally-estimated multivariate predictive functions after inclusion of 942 subjects with incomplete baseline and follow-up measurements using multiple imputations

	SAHS		ARIC		Framingham*	
Risk factor at baseline	Locally-estimated (95% CI)	***Published***	Locally-estimated (95% CI)	***Published***	Locally-estimated (95% CI)	***Published***
Age (years)	***-0.002 (-0.018; 0.014)***	***0.028***	**-0.014 (-0.031; 0.003)**	***0.0173***	NA	NA
Gender	***0.182 (-0.207; 0.570)***	***0.661***	NA	NA	NA	NA
Ethnicity	0.371 (0.024; 0.719)	0.412	0.412 (0.064; 0.759)	0.4433	NA	NA
FPG (mg/dl)	0.094 (0.055; 0.134)	0.079	0.080 (0.056; 0.104)	0.0880	NA	NA
IFG	NA	NA	NA	NA	1.693 (1.276; 2.110)	1.98
SBP (mm Hg)	0.012 (0.001; 0.023)	0.018	0.011 (0.000; 0.022)	0.0111	0.584 (0.215; 0.953)	0.50
Triglyceride(mg/dl)	NA	NA	***0.000 (-0.001;0.001)***	***0.0027***	0.577 (0.179; 0.976)	0.58
HDL (mg/dl)	-0.021 (-0.039;-0.004)	-0.039	-0.012 (-0.029; 0.005)	-0.0122	***0.303 (-0.051; 0.656)***	***0.94***
BMI (kg/m^2^)	0.080 (0.057; 0.104)	0.070	NA	NA	NA	NA
BMI (overweight)	NA	NA	NA	NA	***0.755 (0.389;1.12)***	***0.30***
BMI (obese)	NA	NA	NA	NA	1.209 (0.689;1.729)	0.92
Waist (cm)	NA	NA	0.045 (0.026; 0.063)	0.0273	NA	NA
Height (cm)	NA	NA	-0.030 (-0.050;-0.011)	-0.0326	NA	NA
Family history of T2DM	0.513 (0.190; 0.837)	0.481	0.541 (0.215; 0.866)	0.4981	0.625 (0.300; 0.949)	0.57

Bold and italicized fonts: Estimates from local models whose 95% confidence intervals do not overlap with the published estimates, i.e., we assumed that the published estimates were the true values in the hypothesis testing)

*Categorical variables are used for the Framingham model with the following rule: fasting glucose level: (1 = 100 - 125 mg/dL, 0 ≤ 100 mg/dL), overweight (1 = BMI ≥ 25 & BMI < 30, 0 = otherwise), obese (1 = BMI ≥ 30, 0 = otherwise), low high density lipoprotein (HDL) (1 = HDL < 40 mg/dL in men or < 50 mg/dL in women, 0 = otherwise), high triglyceride level (1 ≥ 150 mg/dL, 0 = otherwise), elevated blood pressure (systolic blood pressure ≥ 130 or diastolic blood pressure ≥ 85 mm Hg, 0 = otherwise)

### Discrimination power

Table 4 compares AUC for the various predictive functions. Although the AUC for all three locally-estimated multivariate models were slightly higher than the corresponding statistic for their *published* counterpart, only in the case of SAHS model did this difference achieve statistical significance. All locally-estimated multivariate models achieved better discrimination power when compared to model that used FPG only (all *P *< 0.001, Table 4), while the locally-estimated Framingham model is the only one that was not statistically better than model that used only 2hPG (*P *= 0.110).

**Table 4:** Comparisons of area under the Receiver Operating Characteristic curve (AUC) for various predictive models evaluated using NHS-92 Cohort

Model 1	AUC (95% C.I)	Model 2	AUC (95% C.I)	*P *value for comparison
SAHS-local	0.857 (0.821;0.894)	SAHS-published	0.839 (0.803;0.874)	0.028
ARIC-local	0.864 (0.829;0.899)	ARIC-published	0.847 (0.812;0.883)	0.065
FRAM-local	0.826 (0.784;0.867)	FRAM-published	0.805 (0.762;0.849)	0.076
SAHS-published	0.839 (0.803;0.874)	ARIC-published	0.847 (0.812;0.883)	0.230
SAHS-published	0.839 (0.803;0.874)	FRAM-published	0.805 (0.762;0.849)	0.028
ARIC-published	0.847 (0.812;0.883)	FRAM-published	0.805 (0.762;0.849)	0.007
FPG-local	0.782 (0.736;0.828)	SAHS-local	0.857 (0.821;0.894)	< 0.001
		ARIC-local	0.864 (0.829;0.899)	< 0.001
		FRAM-local	0.826 (0.784;0.867)	0.047
OGTT-local	0.778 (0.727;0.828)	SAHS-local	0.857 (0.821;0.894)	0.004
		ARIC-local	0.864 (0.829;0.899)	0.002
		FRAM-local	0.826 (0.784;0.867)	0.110
FPG-local	0.782 (0.736;0.828)	SAHS-published	0.839 (0.803;0.874)	< 0.001
		ARIC-published	0.847 (0.812;0.883)	< 0.001
		FRAM-published	0.805 (0.762;0.849)	0.323
OGTT-local	0.778 (0.727;0.828)	SAHS-published	0.839 (0.803;0.874)	0.021
		ARIC-published	0.847 (0.812;0.883)	0.011
		FRAM-published	0.805 (0.762;0.849)	0.371

Out of the three *published* functions, the ARIC model had the highest discrimination power (AUC = 0.847), followed by the SAHS model (AUC = 0.839) and the Framingham model (AUC = 0.805). The performance of the *published* SAHS and ARIC models were not statistically different (*P *= 0.230), but both models had significantly higher discrimination power than the Framingham model (*P *= 0.028 and 0.007, respectively). More importantly, the *published* SAHS and ARIC models were statistically better at discriminating T2DM cases from non-cases when compared to the *local* model that used FPG only (*P *< 0.001) or 2hPG only (*P *= 0.021 and 0.011, respectively).

**Figure 2:**
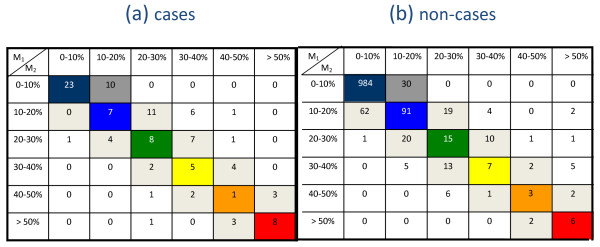
**Comparisons of risk classifications for subjects in NHS-92 cohort using recalibrated ARIC (M_1_) and SAHS (M_2_) models**.

The NRI statistic revealed that overall, the *published* ARIC model was only marginally better than the *published* SAHS model (NRI = 0.127, *P *= 0.060). When we looked at cases and non-cases separately, the ARIC model was not significantly better than the SAHS model in terms of reclassifying cases (Figure 2). Specifically, compared to the SAHS model, 32 cases were appropriately reclassified by the ARIC model at a cost of 21 cases being reclassified inappropriately (NRI = 0.100, *P *= 0.131). However, the ARIC model was better at reclassifying non-cases. In total, 110 non-cases were appropriately reclassified using the ARIC model, at a cost of 76 non-cases being reclassified inappropriately (NRI = 0.026, *P *= 0.013).

### Calibration

The calibration inspections revealed that the *local* models showed good calibration properties (Table 5). In particular, the H-L statistics for *local* models are all less than 11.5 and the predicted incidence rates under all *local* models agree well with observed incidence rates over the 13-year period in the NHS-92 cohort, which was 7.8%. However, the three *published* models showed poor calibration, with the Framingham model being the worst. In particular, the SAHS and ARIC *published* models overestimated the incidence rates, while the Framingham model underestimated the incidence rates. Specifically, the estimated incidence rates from the SAHS and ARIC models were 13.5% and 9.8%, respectively. Meanwhile, estimated incidence rates from the Framingham model is 2.0%.

**Table 5:** Calibration quality of various predictive models evaluated using NHS-92 Cohort (N = 1,401)

Model	HL-statistic	Predicted incidence (%)
SAHS-local	3.52	7.8
ARIC-local	2.28	7.8
FRAM-local	1.45	7.8
FPG-local	3.08	7.8
OGTT-local	3.90	7.8
SAHS-published	48.33	13.5
ARIC-published	14.71	9.8
FRAM-published	264.89	2.0
*Recalibrated *SAHS-published	23.19	11.2
*Recalibrated *ARIC-published	10.31	8.5
Recalibrated FRAM-published	103.15	16.9

**Figure 3:**
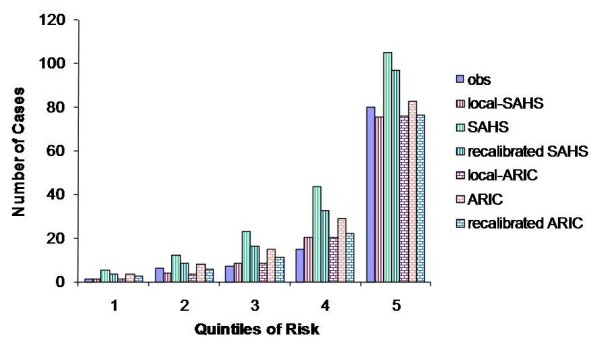
**Observed and predicted number of cases in the different risk quintiles**.

Recalibration improved the calibration quality of ARIC model (Figure 3), but the same cannot be said for the Framingham model. The recalibration procedure seemed to work reasonably well for the SAHS model for subjects in the lowest three quintiles (Figure 3); however, the SAHS model still overestimated the number of cases in the two highest quintiles even after recalibration. The poor performance of the Framingham model could be due to the fact that the Framingham cohort used to derive the model consists almost exclusively of one race while the Singapore population consists of three races with Chinese being different from Malays and Indians. To investigate this possibility, we performed local fitting and recalibration of the three *published* models separately in the Chinese and non-Chinese populations, with the race terms removed from the ARIC and SAHS models. In the Chinese population, the H-L statistic for locally-fitted ARIC, Framingham and SAHS models is 4.22, 3.43 and 1.38 respectively, indicating good calibration properties. However, only recalibrated ARIC and SAHS models show acceptable calibration quality with H-L statistic of 7.31 and 2.23 respectively. The recalibrated Framingham model still shows poor calibration quality (H-L statistic = 26.12). Among non-Chinese population the story is very similar. The H-L statistic for locally-fitted ARIC, Framingham and SAHS models is 1.53, 2.74 and 3.34 respectively. Among the recalibrated *published* models, only ARIC shows acceptable calibration quality with H-L statistic of 6.87. The recalibrated Framingham and SAHS have poor calibration quality with H-L statistic of 19.29 and 140.17, respectively. Thus, the poorer performance of the Framingham model is unlikely only due to differences in race effects between the Framingham cohort and Singapore population. It is more likely that differences in the effect sizes of some of the risk factors also contribute to the poor performance.

## Discussion

Studies in the United States [8,9] and Asia [10] have shown that a multivariable predictive model that incorporated clinical risk factors and fasting blood tests performed better than 2hPG in predicting the risk of T2DM and could replace the OGTT in identifying individuals at increased risk of T2DM that would be suitable candidates for a diabetes prevention program. Our study confirms these findings.

We also found that externally-developed predictive models showed good discriminative power, even when applied to Asian ethnic groups. However, we noted that this did not apply equally to all predictive models. In our population, the discriminatory power of the *published* Framingham model [14] was not statistically better than risk prediction using FPG or 2hPG. Using a multiethnic US cohort, Mann et al. [15] also found that the performance of the Framingham model was significantly worse than ARIC and SAHS models. This may relate to the use of categorical variables in the Framingham model, compared to the use of continuous variables in the other models. Using NRI statistic, we also found that the *published* ARIC model was significantly better than the SAHS model in classifying subjects with low risk of developing T2DM. This was in contrast to the findings of Mann et al., who found the SAHS and ARIC models to be equivalent in terms of discriminative power. The reason that recalibration did not work very well for the SAHS model here was because the *published* model included gender effect, while, in our local population, we did not find differences between gender. The fact that discrepancy in gender effect was causing unsatisfactory recalibration performance was more apparent when we evaluated the calibration quality separately for females and males (Table 6). The recalibration worked well for the SAHS model among males where gender effects did not exist (i.e. male being the reference category in the model), while recalibration did not work well for females.

**Table 6:** Hosmer-Lemeshow (HL) statistics for various published predictive models evaluated using Female and Male subsets of NHS-92 cohort

Model	Female (N = 732)	Male (N = 669)
SAHS-published	37.31	16.25
ARIC-published	7.41	14.17
FRAM-published	112.90	157.66
Recalibrated SAHS-published	23.06	7.11
Recalibrated ARIC-published	5.43	12.25
Recalibrated FRAM-published	53.35	51.22

The validity of externally-developed predictive models depends on the homogeneity of effect size, risk factors distribution and length of follow-up between the two populations. Differences in terms of the last two factors can be remedied using recalibration. Heterogeneity of effect sizes, however, is harder to remedy, and, when it exists, it tends to downgrade the performance of the externally-developed functions. In our case, we initially found a greater effect of baseline FPG and obesity (whether assessed using BMI or waist circumference) in our Asian cohort when compared to the US cohorts that were used to develop the various predictive functions. Other authors have previously reported findings that BMI underestimated the degree of adiposity in Asians as compared to Caucasian populations [23,24]. While we cannot exclude this possibility, our data suggests that these discrepancies may also be caused, to some extent, by the failure of our initial analyses to include those subjects without complete baseline data or FPG at follow-up. After the inclusion of these subjects via multiple imputation, the differences in the parameter estimates were no longer statistically significant. This highlights the importance of assessing dropout effects and avoiding the sole use of subjects with complete measurements when developing and comparing predictive functions. This is because subjects excluded from complete-case analysis often have very different characteristics from those included. Using multiple imputation is a reasonable approach for including individuals with incomplete data. However, multiple imputation does implicitly assume that the probability of individuals having incomplete data does not depend on the missing variable itself (in this case, FPG at follow-up), but, rather, it depends on a set of other variables that are observed. This mechanism of 'missingness' is called *missing at random (MAR)*. The assumption is not testable, but it can be made more plausible by using a more inclusive strategy in which more variables are included in the imputer model for prediction of missing values. In our analysis, we used this strategy by including as many baseline variables that we thought may be good predictors of FPG at follow-up as possible. This strategy seems to work reasonably well, since the coefficients in the local models were more comparable to the published estimates, after multiple imputation. However, given that the amount of imputed data is substantial, we can never completely rule out the possibility of remaining bias caused by differences in the unobserved/unmeasured characteristics that are also associated with FPG at follow-up.

The *published* ARIC and SAHS models included binary 'race' variables in their models. The 'race' coefficient likely represents unique contributions from genetics and socio-economic factors in the external populations used to build the models, and, generally speaking, its validity needs to be examined carefully when it is applied to completely different populations. The assumption of having exactly two race groups in the models is also potentially problematic when the models are to be applied into different populations. Even when the external population has exactly two major race groups, without local fitting of the models, it is not necessarily obvious which group should be regarded as the lower risk group. If more than two race groups (such as in this manuscript), without local fitting it is not necessarily clear which groups to merge to get down to exactly two groups. *We propose that when sufficient local data are available the coefficients for 'race' terms need to be re-estimated using local data. This will avoid assumptions that there are exactly two race groups in the local population and the magnitudes of the race terms and the difference between the races are similar in this population as in the populations used to derive the ARIC and SAHS models. When there are not enough local data, we suggest a two-step approach for risk prediction is used: at the first step, the risks prediction is done by removing the race terms in the models, followed by the second step where the predicted risks are recalibrated within each race (ethnic group). By removing the race terms the relative ordering of risks should be reasonable but the absolute risks are unlikely to be predicted accurately. The recalibration step is needed to ensure that the discrepancy in the absolute risks due to differences in race coefficients is minimized*.

The use of 'age' coefficient should also be considered carefully when the age distribution in the population to which the models were to be applied is very different from the population in which the model was built. In our case, the average age of our subjects is significantly younger than the ARIC and SAHS cohorts. If there is a birth cohort effect or nonlinear age effect, this may explain why we failed to discover significant age effects in our *local* models. Indeed, when sufficient local data is available, performing recalibration for different age groups separately in a similar manner to the ethnic-specific recalibration above is arguably better than blindly extrapolating the age effects.

In addition, we also would like to draw attention to the heterogeneity in the T2DM definition employed by the different models. Ideally, T2DM is defined based on outcomes of both FPG and OGTT. However, due to lack of measurements, only the SAHS study used this definition consistently to derive its model. The Framingham model used FPG/OGTT to define T2DM at baseline; however, it relied only on FPG to identify incident T2DM at follow-up, while the ARIC model failed to incorporate OGTT when identifying T2DM cases at baseline. In our dataset, we used FPG/OGTT at baseline, but only FPG measurements were available to define incident T2DM at follow-up. While investigations using our FPG and OGTT data at baseline showed that the misclassification rates were not statistically related to ethnicity or gender (data not shown), the possibility of differential misclassification in which subjects with certain profiles is more likely than others to be diagnosed with T2DM using OGTT only, is real. Differential misclassification will likely affect the recalibration performance and thus potentially make prediction of absolute risk less reliable. However, we believe that predictive risk would still work adequately well for the purpose of classifying individuals into high-risk and low-risk groups.

## Conclusions

In summary, we have confirmed that multivariate predictive functions based on clinical and biochemical measurements made in the fasting state outperform an OGTT for predicting future T2DM. In addition, we have shown that the effects of risk factors on the risk of incident T2DM are broadly similar in Asians as they are in other ethnic groups. This means that in populations where data from relatively large and well-documented prospective cohort needed for developing *local* predictive function is not available, the use of a predictive function derived in another population is feasible, and, arguably preferable, to using FPG or OGTT alone. The only requirement for the function to be applicable in another population would be the availability of a small, pilot cohort in the order of several hundred subjects, from which survival rates and average risk factors can be estimated and used for recalibration. Our data further suggests that if one were to choose from the three predictive functions tested, the ARIC predictive function should be the choice.

## Competing interests

The authors declare that they have no competing interests.

## Authors' contributions

CC wrote the original version of manuscript and contributed to statistical analyses. SM and DH contributed to supervising the implementation of the National Health Survey (NHS) and the subsequent SP2 study. EST conceived the SP2 study and contributed to the Results and Discussion sections. JL conceived the SP2 study and contributed to the Discussion section. MT contributed to the statistical analyses and maintained the quality of the survey database. EHSC contributed to the statistical analyses and wrote part of the Method section. AS directed and supervised the overall quality of statistical analyses and contributed to all parts of the manuscripts. All authors have read and approved the submitted manuscript.

## Appendix

### Appendix A: Recalibration Method

Several authors e.g. [25,26] have suggested the use of the following formula in order to recalibrate predicted probability computed using models developed in an external population,

(1) $$\text{p}\left(\underline{\text{x}}\right)=\text{1}-\text{S}\left(\underline{\text{m}}\right)\text{exp}\left({\left(\text{x}-\underline{\text{m}}\right)}^{\prime }\underline{\beta }\right)$$

where p(**x**) is the recalibrated probability of an event for subjects with risk factors **x**, S(**m**) is the survival rates for subjects with average risk factors **m **in the local population (in our case, the NHS-92 cohort) and **β **the vector of regression coefficients estimated from the external population. This recalibration formula can be expected to work satisfactorily when there are no or little differences between the risk factors and their magnitude in the external and local populations.

The recalibration formula was originally suggested to recalibrate Framingham CHD risk [25], where **β **is estimated using Cox proportional hazard model; however, for interval-censored data with relatively low incidence rates, **β **from logistic regression can also be used. The justification for this is because for interval-censored data, Cox proportional hazard model is the same as specifying complementary log-log regression model, which, in turn, is very similar to the logistic regression model when incidence rates are low.

## Pre-publication history

The pre-publication history for this paper can be accessed here:

http://www.biomedcentral.com/1471-2288/12/48/prepub
